# S100A8 promotes the proliferation, migration and invasion in bladder cancer cells

**DOI:** 10.7150/jca.102201

**Published:** 2025-01-01

**Authors:** Hao-ran Xu, Zhen Pang, Qin-zhang Wang, Song Ou-Yang

**Affiliations:** 1 Department of Urology, The First Affiliated Hospital of Shihezi University, Shihezi, Xinjiang, China.; 2 Clinical Research Center for Urinary System Diseases of Xinjiang Production and Construction Corps, Shihezi, Xinjiang, China.

**Keywords:** S100 calcium-binding A8, bladder cancer, proliferation, migration, invasion

## Abstract

**Background:** There is mounting evidence that S100 calcium-binding A8 (S100A8) is involved in inflammation and cancer. However, whether S100A8 promotes the proliferation, invasion and migration of bladder cancer (BC) is still not completely clear. To investigate the influence of S100A8 on the proliferation, migration and invasion of BC.

**Methods:** Based on Gene Expression Omnibus (GEO) and The Cancer Genome Atlas (TCGA) databases, genes related to the grading, staging, proliferation, migration and invasion of BC were screened, and S100A8 was selected as the target gene for further studies. Immunohistochemical staining were employed to examine the protein expression levels of S100A8 on adjacent tissues and BC tissues. The gene expression level of S100A8 in Pan cancer cell lines was analyzed through the Cancer Cell Line Encyclopedia (CCLE) database, and the HT-1376 cell line was selected for subsequent experiments. Overexpression recombinant lentivirus and short hairpin RNA-encoding lentivirus were used to overexpress and knock down S100A8 in HT-1376 cells via infection. The mRNA and protein expression levels of S100A8 were detected by reverse transcription-quantitative PCR and western blotting. The proliferation of BC cells was analyzed using Cell Counting Kit-8 and 5-ethynyl-2'-deoxyuridine assays. The Wound‑healing assay and Transwell assay were used to estimate the migration and invasion of BC cells.

**Results:** The results demonstrated that S100A8 was highly expressed in BC based on the GEO and TCGA databases. In addition, compared with those of HT-1376 cells and the negative control group, the proliferation, migration and invasion of S100A8-overexpressing HT-1376 cells were enhanced, while those of S100A8-knockdown HT-1376 cells were reduced. Furthermore, S100A8 was differentially expressed in non-muscle invasive BC and muscle invasive BC, and in low- and high-grade BC.

**Conclusions:** In this study, the bioinformatics and in vitro experiments revealed that S100A8 could promote the proliferation, invasion and migration of BC cells. Consequently, S1008A emerges as a promising diagnostic and therapeutic target for BC.

## Introduction

Bladder cancer (BC) is a widespread neoplastic disease, mainly originating from the bladder lining, with ~75% arising from the urothelium, presenting as pure urothelial carcinoma, and histological variations accounting for the remaining 25% 1,2. In 2020, there were 523,278 new cases and 212,536 deaths worldwide 3. The prevalence and mortality rates in men are 3-4 times those in women, and both morbidity and mortality of BC are second only to prostate cancer among urinary system tumors 3. An estimated 92,900 new cases and 41,400 deaths occurred in China in 2022. Among the new cases, 73,200 were diagnosed in men and 19,700 were diagnosed in women. In addition, there were 32,500 deaths in men and 8,800 deaths in women 4. BC ranks eighth in terms of both age-standardized incidence rate and age-standardized mortality rate 4. BC can be classified as both high- and low-grade cancer, and can also be divided into muscle invasive BC (MIBC) and non-muscle invasive BC (NMIBC) 5-7. NMIBC accounts for ~75% of new-onset BC cases 8. Patients with NMIBC have a longer survival period and a lower risk of tumor-specific death than those with MIBC 9. Compared with low-grade cancer, high-grade cancer is more likely to invade the muscularis and is associated with a higher mortality rate 9. However, NIMBC has a 70% recurrence rate and 30% of NMIBC can progress to muscle-invasive disease after initial treatment. Recurrence and progression to high-grade cancer may lead to poor prognosis 9. Early diagnosis is essential for effective treatment and good prognosis of patients 10. The gold standard for the positive diagnosis of BC is cystoscopy, but its invasiveness hinders its early use and non-invasive diagnostic markers are required 11. Urine cytology is the most cost-effective urine-based method for the diagnosis of high-grade BC 12. Notably, the sensitivity of any cytological classification method for low-grade BC is lower than that for high-grade BC due to the closer similarity between low-grade lesions and normal cellular morphology 13. In the past, much effort has been invested in developing protein- and molecule-based urine tests to diagnose BC, such as bladder tumor antigen and urine nuclear matrix protein 22 tests 14,15. Although a number of these tests are more sensitive than urine cytology in detecting BC, they are usually limited by lower specificity, false-positive results and better utility in high-grade lesions 16,17. Although transurethral resection of bladder tumor is the standard of care for the treatment of NMIBC, it is a major surgery requiring formal anesthesia, which could be a burden for patients with recurring diseases 18. Immunotherapy has been applied to patients with bladder cancer in the form of Mycobacterium bovis bacillus Calmette Guerin (BCG), which is still the first-line treatment for non-muscle invasive disease 19. However, the remarkable results achieved with checkpoint inhibitor drugs, including pembrolizumab and atezolizumab, have driven the exploration of optimizing these and other forms of non-muscle invasive and advanced bladder cancer immunotherapy 19. Radical cystectomy is a standard of care in localized MIBC 20. To sum up, it is vital to find a valid biomarker to predict the staging, grading, diagnosis and prognosis of BC.

In 1956, S100 family proteins were found in the brain, and were referred to as S100 proteins because they are soluble in 100% ammonium sulfate at neutral pH 21. One of these is S100 calcium-binding A8 [S100A8; also called myeloid-related protein (MRP)-8 or calgranulin-A], which binds to S100 calcium-binding protein A9 (S100A9; also called MRP-14 or calgranulin-B) to form polymers, which serve a role in inflammation and cancer 22. S100A8/A9 promotes inflammatory responses to protect the body from pathogens such as bacteria, viruses and fungi. For example, after being released at the site of infection, it induces a host defense function locally and exhibits a wide range of antifungal and antibacterial potential 23,24. The expression of S100A8 and S100A9 is tissue- and cell-specific and is changeably enhanced in various cancer types and inflammation-related diseases. Recent studies have demonstrated that S100A8 and S100A9 can stimulate the proliferation, migration and invasion of most cancer types 25-27. For instance, S100A8 activates the NF-κB pathway, and then blocks BC cell apoptosis 28. S100A8 can activate the Toll-like receptor 4 (TLR4)-NF-κB signaling pathway, which upregulates VEGF expression, and thus, leads to the invasion and metastasis of cholangiocarcinoma cells 29. In addition, S100A8/A9 may also participate in the invasion of pancreatic cancer cells 30.

However, whether S100A8 promotes the proliferation, invasion and migration of BC is still not completely clear. Therefore, the present study explored the proliferation, invasion and migration of S100A8 in bladder cancer cells.

## Materials and Methods

### Public data access

All public bioinformatics data were obtained from The Cancer Genome Atlas (TCGA) database and Gene Expression Omnibus (GEO) database. The differentially expressed genes were screened from the GEO database, in which the keyword were bladder cancer. Finally, three groups (GSE13507, GSE31684 and GSE120736) were selected, which can simultaneously meet the requirements of grouping MIBC and NMIBC, low-grade and high-grade bladder cancer, and were used for subsequent screening and analysis of candidate differential genes. Next, the intersection of expressed genes with significant differences was analyzed. In addition, The R software package TCGAbiolinks version 2.21.1 was used to capture the transcripts per kilobase of exon model per million mapped reads (TPM) of the TCGA-BLCA dataset in real time to screen for superficial BC transitional cell carcinoma of bladder, the invasion of bladder cancer or not, the stage of BC and so on. Next, the R language was used to batch calculate the S100A8 gene, calculate the Spearman correlation coefficient between S100A8 and all mRNA genes, and sort all gene correlation coefficients of S100A8 gene from high to low. Finally, the Gene set enrichment analysis (GSEA) model of Kyoto Encyclopedia of Genes and Genomes (KEGG) pathways was constructed by using the R software package clusterProfiler version 4.11.0. GSEA of S100A8 was performed by analyzing RNA-sequencing data from the BC dataset in TCGA.

### Cell lines and culture

The SV-HUC-1 normal urothelial cell line (Procell Life Science & Technology Co., Ltd.) and the HT-1376 BC cell line (Annoron Life Science & Technology Co., Ltd.) were used in the present study. HT-1376 cells were cultured in Minimum Essential Medium (Biochannel Science & Technology Co., Ltd) supplemented with 10% FBS (Shanghai ExCell Biology, Inc.) and 1% penicillin-streptomycin (Biosharp Life Sciences). SV-HUC-1 cells were cultured in Hams F-12K medium (Procell Life Science & Technology Co., Ltd.) supplemented with 10% FBS and 1% penicillin-streptomycin. The aforementioned cells were cultured at 37˚C in a 95% air and 5% carbon dioxide humidification incubator.

### Tissue specimens

Paraffin section of bladder cancer diagnosed by pathology of The First Affiliated Hospital of Shihezi University (Shihezi, China) between January 2021 and January 2023 were collected. 46 pairs of BC tissues and adjacent non-tumor tissues were collected from 46 patients, aged 65.16 ± 15.17 years, ranged from 30-94 years. Patients were included when: i) they had primary BC, excluding recurrence cases; ii) they did not receive radiotherapy, chemotherapy or other neoadjuvant treatment; iii) their diagnoses were confirmed by professional pathologists according to the BC histopathological diagnostic criteria; and iv) had no history of other malignant tumors. A total of 46 patients were included, including 6 female and 40 male patients. Among them, 28 were above 60 years old and 18 were below 60 years old. The diagnosis of patients indicated 25 cases of low-grade BC and 21 cases of high-grade BC. According to another staging method, patients were also divided into 26 NMIBC cases and 20 MIBC cases. The present study was conducted with approval from the Ethics Committee of The First Affiliated Hospital of Shihezi University (ethics committee approval no. KJ2021-277-01; Shihezi, China). All of the patients signed an informed consent form.

### Immunohistochemistry

Formalin‑fixed paraffin‑embedded sections of the tumor tissues and each adjacent normal tissue were sliced to a thickness of 4 µm and placed on slides coated with poly‑L‑Lysine (Shenzhen Boshida Optical Instrument Co., Ltd.) The paraffin sections containing pathological tissues were dried in an oven at 65˚C for 40 min, and were subsequently dewaxed with xylene and descending gradient ethanol. The tissue sections were microwaved in 10 mM sodium citrate buffer solution (pH 6.0; Wuhan Servicebio Science & Technology Co., Ltd.) at 95˚C for 10 min. After antigen retrieval was completed, the sections were cooled in citrate buffer at room temperature for 20 min. The endogenous peroxidase activity was then inactivated by 3% hydrooxidase (Shandong Anjie high tech Technology Co., Ltd) at room temperature for 20 min. Next, the tissues were covered with 3% Bovine Serum Albumin (BSA) dropwise and blocked for 30 min at room temperature. The sections were incubated overnight at 4˚C with primary antibodies, namely rabbit anti-S100A8 antibody (1:1,000; Abcam; no. ab288715). After the sections were incubated at room temperature for 20 min with the goat anti-rabbit (1:2000; Wuhan Servicebio Science & Technology Co., Ltd., no. GB23303), 3,3'-diaminobenzidine was added to the sections for 1 min, and subsequently, sections were counterstained with hematoxylin at room temperature for 3 min. The staining intensity was scored as follows: 0, blue, negative; 1, light yellow, weakly positive; 2, brown or dark yellow, moderately positive; or 3, dark brown, strong positive. The proportion of stained cells was scored as follows: 1, ≤25% of cells stained; 2, 26-50% of cells stained; 3, 51-75% of cells stained; or 4, ≥75% of cells stained. The staining score was the product of the staining intensity score and the proportion of stained cells score. Tissue section scores ≤1 indicated negative expression, scores of 2-4 indicated weak positive expression, scores of 5-8 indicated moderate positive expression and scores of 9-12 indicated strong positive expression. The stained sections were observed with a microscope (Nikon Corp.).

### Lentivirus infection and stable cell lines

Lentiviral infection was performed when the cell confluence was 50%. The S100A8-overexpressing lentiviruses (Ubi-MCS-3FLAG-SV40-EGFP-IRES-puro) and short hairpin RNA (shRNA)‑encoding lentiviruses (hU6-MCS-CBh-gcGFP-IRES-puro), with the target sequence 1 TCAACACTGATGGTGCAGTTA and target sequence 2 GTGTCCTCAGTATATCAGGAA, which were purchased from Shanghai GeneChem Co., Ltd., were used for lentivirus infection. At the time of transfection, the cells were incubated with the Minimum Essential Medium containing lentivirus with multiplicity of infection (MOI) of 80 at 37˚C in a 95% air and 5% carbon dioxide humidification incubator for 12 hours, and then, the cells were cultured with in Minimum Essential Medium supplemented with 10% FBS and 1% penicillin-streptomycin containing 5 µg/ml puromycin for two weeks for subsequent experiments.

### Western blotting

RIPA lysis buffer (Beijing Solarbio Science & Technology Co., Ltd.) containing 1% phenylmethanesulfonyl fluoride (Beijing Solarbio Science & Technology Co., Ltd.) and 1% protein phosphatase inhibitor was used to lyse cells on ice. Ultrasound and 12,000 rpm at 4˚C for 15 min centrifugation were used to extract total protein. Based on the protein concentration determined using the BCA protein detection kit (Vazyme Biotech Co., Ltd.). Each group of protein samples was adjusted to the same quality by adding RIPA lysis buffer and loading buffer solution (Biosharp Life Sciences). The 20 µg protein samples, which were loaded in each lane, were separated by 15% SDS-PAGE, and subsequently transferred to polyvinylidene difluoride membranes (MilliporeSigma) at 300 mA for 60 min. Subsequent to blocking with 5% skim milk powder for 2 h at room temperature, the membranes were incubated overnight at 4˚C with primary antibodies, including rabbit anti-human S100A8 antibody (1:1,000; Abcam; no. ab288715) and mouse anti-human β-actin (1:1,000; ZSGBBIO Technologies, Inc; no.TA-09.). After the corresponding secondary antibodies, including goat anti-rabbit (1:10,000; Abcam; no. ab205718) and goat anti-mouse (1:10,000; ZSGBBIO Technologies, Inc; no. ZF-0312.) secondary antibodies, were added at room temperature for 2 h, the protein bands were visualized using the enhanced chemiluminescence kit (Biosharp Life Sciences). The films were developed in a dark room, scanned, and analyzed using the ImageJ Software (Version 1.53).

### Reverse transcription-quantitative PCR

To collect cells total RNA, the Fast Pure Cell/Tissue Total RNA Isolation Kit (Vazyme Biotech Co., Ltd., RC101-01.) was used. Cells total RNA concentrations were determined using the NanoDrop 2000 (Thermo Fisher Scientific, Inc.). Subsequently, cDNA was generated by reverse transcription of RNA using a HiFiScript cDNA Synthesis Kit (CoWin Biosciences, CW2569M). The conditions for reverse transcription were 42 ° C for 15 min and 85 ° C for 5 min. UltraSYBR Mixture (CoWin Biosciences, CW2601M) was utilized to analyze RNA expression. PCR was conducted at 95˚C for 10 min followed by 40 cycles of 95˚C for 15 sec and 60˚C for 60 sec. Gene expression was normalized to β-actin, and calculated using the 2-ΔΔCq method 31. Each independent experiment was repeated three times. RT-qPCR was carried out with the following primers: S100A8 forward, 5'-CCTGAAGGTTCTGTTTTTCAGGT-3' and reverse, 5'-GGTCATCCCTGTAGACGGCA-3'; and β-actin forward, 5'-AGTGTGACGTTGACATCCGTA-3' and reverse, 5'-CCAGAGCAGTAATCTCCTTCT-3'.

### Cell Counting Kit-8 assay

Transfected and untransfected HT-1376 cells were seeded into a 96-well plate, with 1×10^4^ cells per well, and incubated in a 37˚C incubator. On days 0, 1, 2, 3 and 4, 10 µl CCK-8 (Dojindo Laboratories, Inc.) and 90 µl serum-free media were added to each well to replace the previous medium. The cells were incubated in a 37˚C incubator for 2 h, and then absorbance was measured using an enzyme labeling instrument at a wavelength of 450 nm to determine cell viability.

### 5-ethynyl-2'-deoxyuridine assay

Transfected and untransfected HT-1376 cells were seeded into a 96-well plate, with 1×10^4^ cells per well, and incubated in a 37˚C incubator. The next day, 50 µl culture medium was discarded and 50 µl EdU solution (APeXBIO Technology LLC) was added to each well, and then the cells were incubated in a 37˚C incubator for 2 h, after which the medium was discarded and 100 µl of 4% paraformaldehyde was added to each well at room temperature for 15 min. Next, the solution was discarded and 100 µl of 0.3% TritonX-100 was added to each well at room temperature for 15 min. Subsequently, the solution was discarded and 100 µl Click reaction solution was added to each well at room temperature for 25 min. Next, the solution was discarded and 100 µl of 5 µg/ml Hoechst 33342 solution was added to each well at room temperature for 15 min, after which images of the cells were captured, and cells were counted in three fields with a fluorescence inverted microscope (Olympus Corp.).

### Wound‑healing assay

When cell confluence reached ~80%, a scratch wound was generated using the tip of 200‑µl pipette by making a straight line and the culture medium was replaced by MEM supplemented with 1% FBS. Digital photographs were obtained at 0 and 24 h after scratching under a microscope at ×100 magnification (Olympus Corp.) and the scratch area was measured using ImageJ software (version 1.46; National Institutes of Health).

### Transwell migration and invasion assays

For the migration assay, 200 µl serum-free medium containing 2×10^4^ transfected and untransfected cells was added to the upper Transwell chambers (Corning, Inc.), and 600 µl of 20% serum (Shanghai ExCell Biology, Inc.) medium was added to the lower chambers. After the cells were placed in the 95% air and 5% carbon dioxide humidification incubator at 37˚C. for 36 h, the cells that had passed through the polycarbonate membrane were fixed for 15 min at room temperature with 4% paraformaldehyde, stained with crystal violet for 15 min at room temperature, imaged and counted in three fields under a fluorescence inverted microscope (Olympus Corp.). For the invasion assay, 200 µl serum-free medium containing 3×10^4^ transfected and untransfected cells was added to the upper Transwell chambers with pre-added 2 h 4˚C Matrigel (BD Biosciences), and 600 µl of 20% serum (Shanghai ExCell Biology, Inc.) medium was added to the lower chambers. After the cells were placed in the 95% air and 5% carbon dioxide humidification incubator at 37˚C. for 48 h, the cells that had passed through the polycarbonate membrane were fixed for 15 min at room temperature with 4% paraformaldehyde, stained with crystal violet for 15 min at room temperature, imaged and counted in three fields under a fluorescence inverted microscope (Olympus Corp.).

### Statistical analysis

Statistical analysis was performed using GraphPad Prism 9.0 (Dotmatics). All experiments were repeated three times. Values from experiments are presented as the mean ± standard deviation. An unpaired two-sided Student's t-test was used to perform comparisons between two groups. one‑way analysis of variance followed by Tukey's post‑hoc test was used to perform comparisons among multiple groups. Differences in S100A8 gene expression between adjacent noncancerous tissues and cancer tissues were examined using a paired t‑test. *P* <0.05 was considered to indicate a statistically significant difference.

## Results

### Results based on the bioinformatics prediction

**Figure 1:**
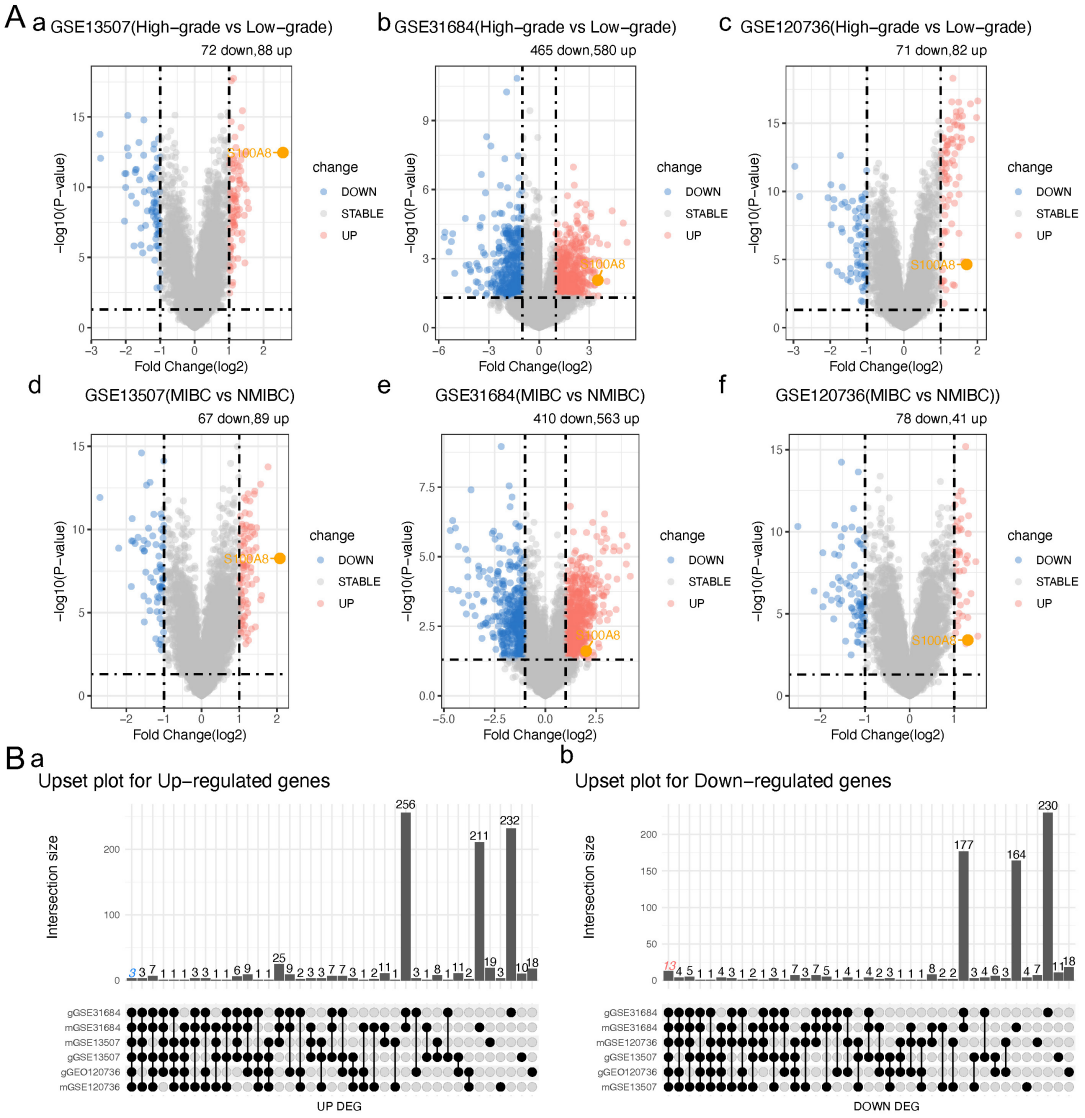
**S100A8 expression in a public BC database.** (Aa) Volcano plot of genes that were differentially expressed in high- and low-grade BC in the GSE13507 dataset. (Ab) Volcano plot of genes that were differentially expressed in high- and low-grade BC in the GSE31684 dataset. (Ac) Volcano plot of genes that were differentially expressed in high- and low-grade BC in the GSE120736 dataset. (Ad) Volcano plot of genes that were differentially expressed in MIBC and NMIBC in the GSE13507 dataset. (Ae) Volcano plot of genes that were differentially expressed in MIBC and NMIBC in the GSE31684 dataset. (Af) Volcano plot of genes that were differentially expressed in MIBC and NMIBC in the GSE120736 dataset. (Ba) Intersection of up-regulated genes. (Bb) Intersection of down-regulated genes. BC, bladder cancer; MIBC, muscle invasive BC; NMIBC, non-muscle invasive BC; S100A8, S100 calcium-binding A8.

To clarify S100A8 expression in BC, MIBC was compared with NMIBC, and high-grade BC was compared with low-grade BC in three BC datasets from the GEO database (GSE13507, GSE31684 and GSE120736). Next, the intersection of expressed genes with significant differences was analyzed. Finally, 16 significantly differentially expressed genes were identified: ANXA10, ST3GAL5, TMPRSS4, CYP3A5, SLC14A1, BTBD16, SPOCD1, CDC42EP5, BCAS1, VSIG2, CAPNS2, FAM3D, HSD17B2, S100A8, CDCA5 and FAM83D. Among them, S100A8, CDCA5 and FAM83D were significantly upregulated, and the remaining genes were significantly downregulated (Table 1 and 2; Fig. 1A and B). The PrognoScan tool was used to perform a meta-analysis of the prognostic value of the sur vival data in the GSE13507 dataset for the overall survival (OS) and disease-specific survival (DSS) of candidate genes in BC. The results showed that the high expression of S100A8 and CDCA5 in bladder cancer was associated with DSS Significantly correlated with poor prognosis of OS (*HR* >1, *P* <0. 05, Table 3). This study will focus on the sub gene S100A8, and other up-regulated and down-regulated genes are also sub genes of this study, which will be the content of subsequent research.

### S100A8 expression in a public BC database and the local cohort

**Figure 2:**
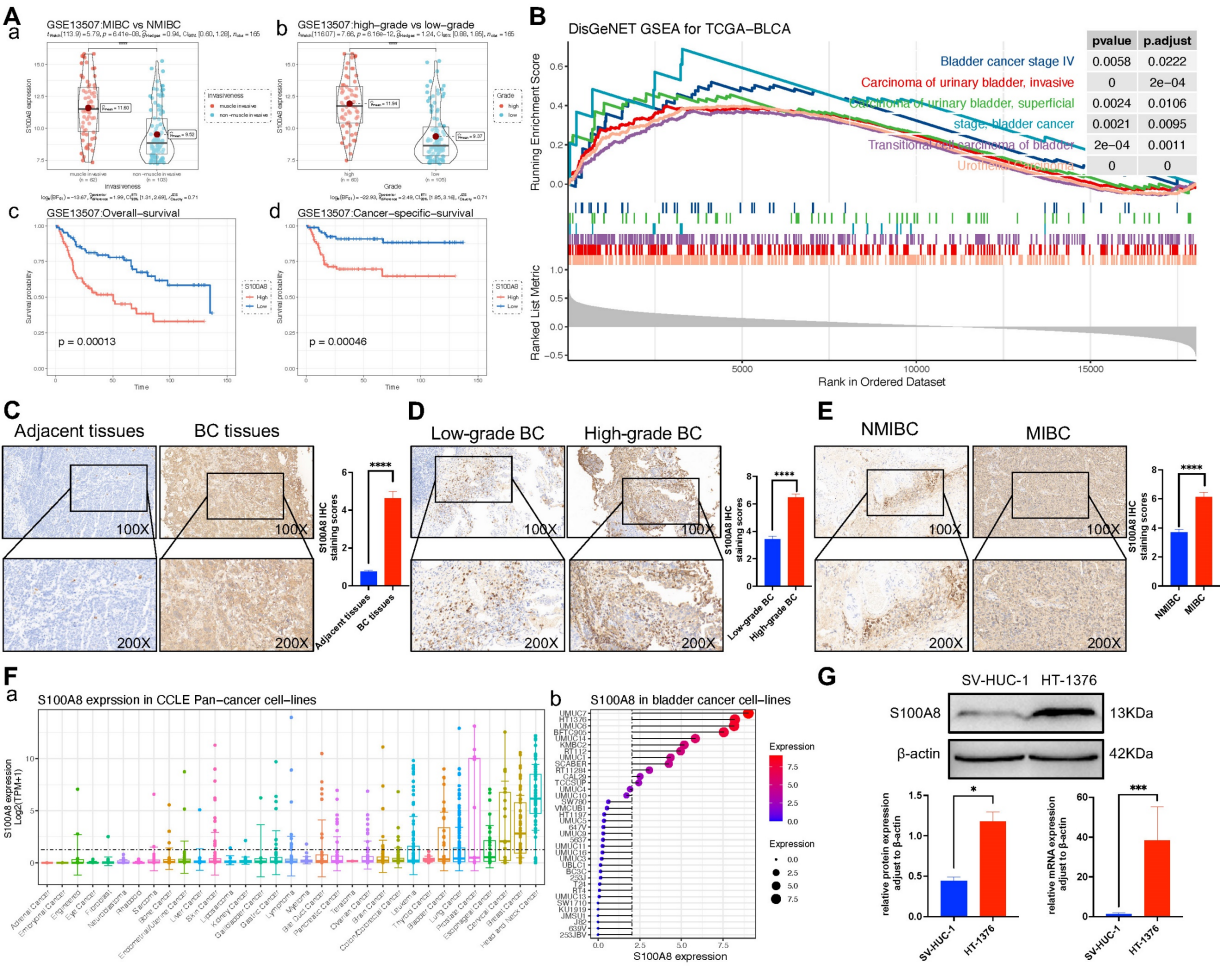
** S100A8 expression in BC tissues and cells.** (Aa) S100A8 expression in MIBC and NMIBC groups in the GSE13507 dataset. (Ab) S100A8 expression in high- and low-grade groups in the GSE13507 dataset. (Ac) Overall survival analysis of S100A8 in the GSE13507 dataset. (Ad) Cancer-specific survival analysis of S100A8 in the GSE13507 dataset. (B) Disease ontology gene set enrichment analysis of S100A8 in TCGA BC dataset. (C) S100A8 expression in BC and adjacent tissues. Score comparison of S100A8 expression in BC and adjacent tissues. (D) S100A8 expression in BC tissues of different stages and grades. Comparison of S100A8 expression scores among groups based on two different grouping methods. (E) S100A8 gene expression levels in Pan cancer cell lines and BC cell lines. (F) S100A8 expression based on western blotting and reverse transcription-quantitative PCR. ^*^P <0.05, ^**^P <0.01, ^***^P <0.001, ^****^P <0.0001. BC, bladder cancer; S100A8, S100 calcium-binding A8.

To evaluate the effect of S100A8 on the proliferation, invasion and migration of BC, the GSE13507 dataset from the GEO database was analyzed and further grouped based on muscle layer invasion, and high and low grades. The results indicated that S100A8 gene expression was significantly higher in MIBC than in NMIBC, and its gene expression was significantly higher in high-grade BC. The survival analysis results implied that the high S100A8 expression group had a lower overall survival and cancer-specific survival than the low S100A8 expression group (Fig. 2A). To further evaluate whether the S100A8 gene has a promoting effect on BC, GSEA of S100A8 was performed by analyzing RNA-sequencing data from the BC dataset in TCGA. The analysis results demonstrated that S100A8 was significantly positively associated with multiple types of BC, such as stage IV BC and MIBC, suggesting that the S100A8 gene may be involved in the proliferation, invasion and migration of BC (Fig. 2B). In addition, paraffin sections of BC tissues and adjacent tissues were collected from 46 patients with BC. The clinicopathological data of 46 patients are shown in Table 2. Next, S100A8 protein expression in BC tissues and adjacent tissues was examined using Immunohistochemistry (IHC). The results revealed high staining scores of the S100A8 protein in BC tissues compared with adjacent tissues (P<0.0001; Fig. 2C). Subsequently, pathological BC tumor tissue specimens were analyzed and further grouped based on muscle layer invasion, and high and low grades. The IHC results showed that the staining scores of the S100A8 protein in the high-grade BC group were higher than those in the low-grade group (*P* <0.01; Fig. 2D). The staining scores of the S100A8 protein were higher in the MIBC group compared with the NMIBC group (*P* <0.0001; Fig. 2E). These results are similar to those obtained from the geo database. In order to screen better BC cell lines for subsequent studies, we analyzed the gene expression level of S100A8 in BC cell lines through Cancer Cell Line Encyclopedia (CCLE) database, and the results showed that the S100A8 expression of bladder cancer cell line HT-1376 was high (Fig. 2F). Then, the differential expression of S100A8 in the HT-1376 BC cell line and the SV-HUC-1 normal urothelial cell line was verified using Western blot (WB) and Reverse transcription-quantitative PCR (RT-qPCR) at the cellular level. The results revealed high S100A8 protein and mRNA expression in HT-1376 cells compared with SV-HUC-1 cells (*P* <0.001; Fig. 2G). These results indicated that S100A8 was highly expressed in BC and was positively associated with tumor stage and grade.

### Effect of S100A8 on viability in BC cells

**Figure 3:**
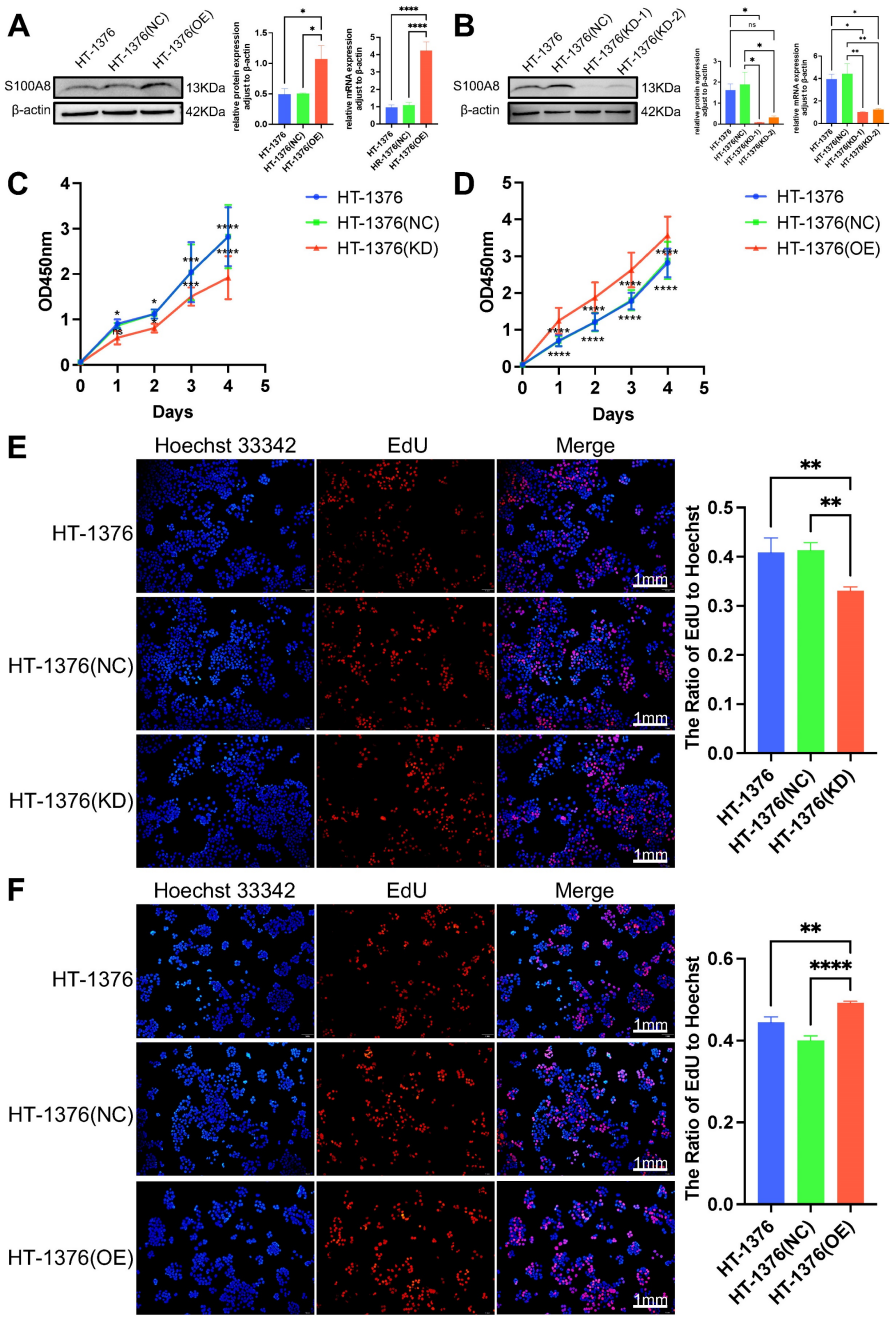
** Effects of S100A8 on viability in HT-1376 cells.** (A) WB and RT-qPCR were used to verify S100A8 protein and mRNA expression after overexpression. (B) WB and RT-qPCR were used to verify S100A8 protein and mRNA expression after knockdown. (C) CCK-8 results of the knockdown, HT-1376 group and NC groups. (D) CCK-8 results in the overexpression, HT-1376 group and NC groups. (E) EdU results for the knockdown, HT-1376 group and NC groups (scale bars: 1 mm). (F) EdU results for the overexpression, HT-1376 group and NC groups (scale bars: 1 mm). *P <0.05, **P <0.01, ***P <0.001, ****P <0.0001. CCK-8, Cell Counting Kit-8; EdU, 5-ethynyl-2'-deoxyuridine; NC, negative control; RT-qPCR, reverse transcription-quantitative PCR; S100A8, S100 calcium-binding A8; WB, western blotting.

To further determine the role of S100A8 in cell proliferation, migration and invasion, S100A8 was overexpressed and knocked down in HT-1376 cells via infection with overexpression recombinant lentivirus (OE group) and shRNA‑encoding lentivirus with two sequences (KD-1 and KD-2 group), respectively. In addition, HT-1376 cells were also infected with a negative control lentivirus as a negative control group (NC group). WB and RT-qPCR were used to verify the infection efficacy. High S100A8 mRNA and protein expression was observed in the OE group compared with the HT-1376 group and NC group (Fig. 3A). The additional results revealed lower S100A8 mRNA and protein expression in both the KD-1 group and the KD-2 group compared with the HT-1376 group and NC group (Fig. 3B), and the knockdown infection efficiency in the KD-1 group was stronger than that in the KD-2 group. Thus, cells from the KD-1 group were used for subsequent experiments. Cell Counting Kit-8 (CCK-8) and 5-ethynyl-2'-deoxyuridine **(**EdU) assays were performed in HT-1376 cells after S100A8 was knocked down or overexpressed. Downregulation of S100A8 inhibited the viability of BC cells (Fig. 3C and E), while overexpression of S100A8 promoted the viability compared with that in the uninfected HT-1376 group and NC group (Fig. 3D and F). These results suggested that S100A8 promoted the viability and proliferation of BC cells.

### Effect of S100A8 on migration and invasion in BC cells

**Figure 4:**
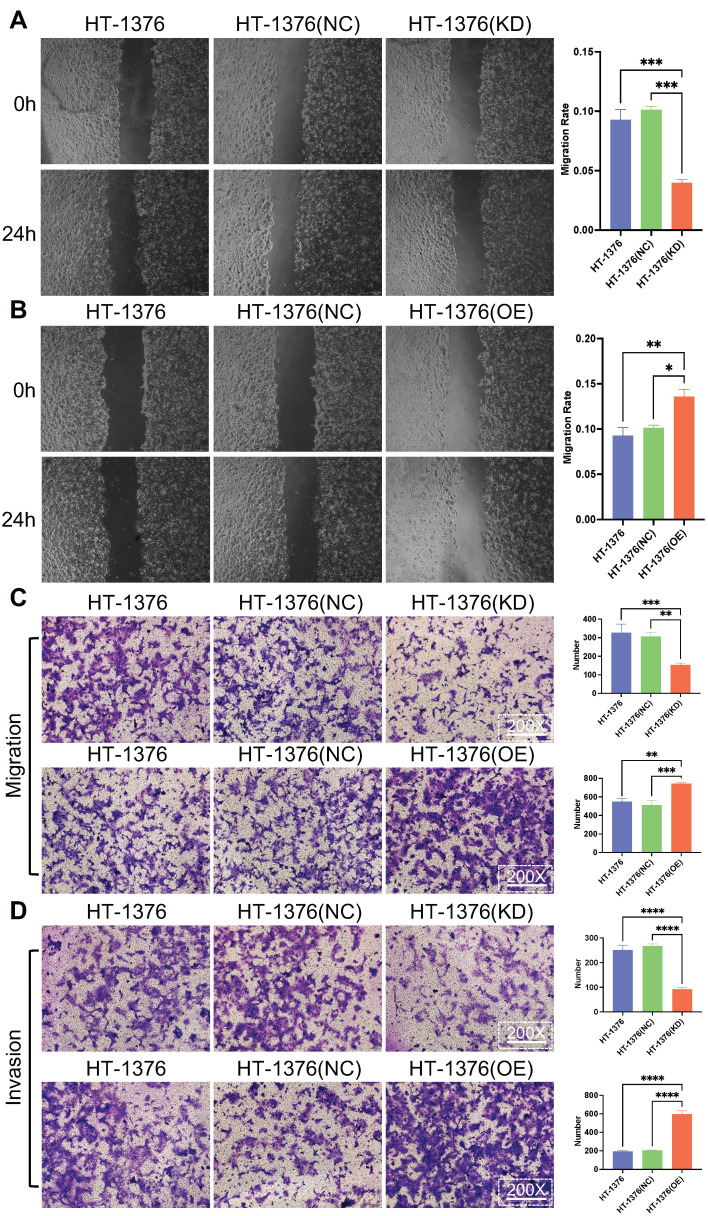
** Effects of S100A8 on migration and invasion in bladder cancer cells.** (A) Wound healing assays were conducted to assess the migratory ability of HT-1376 cells after S100A8 knockdown. (B) Wound healing assays were conducted to assess the migratory ability of HT-1376 cells after S100A8 overexpression. (C) Effects of knockdown and overexpression of S100A8 on migration in a Transwell assay (magnification, ×200). (D) Effects of knockdown and overexpression of S100A8 on invasion in a Transwell assay (magnification, ×200). ^*^P <0.05, ^**^P <0.01, ^***^P <0.001, ^****^P <0.0001. S100A8, S100 calcium-binding A8.

It was ascertained whether the change in S100A8 expression affected the migration and invasion of BC cells. Wound healing assays were used for cell migration assessment. The results indicated that the migratory ability of HT-1376 cells was inhibited due to S100A8-knockdown, while the migration of HT-1376 cells was promoted after the transfection of S100A8-overpression (Fig. 4A and B). Transwell migration and invasion assays were performed in HT-1376 cells after S100A8 was knocked down or overexpressed. Downregulation of S100A8 led to reduced migration, while overexpression of S100A8 promoted migration compared with that of the HT-1376 group and NC group (Fig. 4C). Consistent with these findings, compared with that of the HT-1376 group and NC group, the invasion of S100A8-overexpressing HT-1376 cells were enhanced, while that of S100A8-knockdown HT-1376 cells were reduced (Fig. 4D). Thus, the present study suggested that S100A8 might promote the migration and invasion of BC cells.

## Discussion

BC is a frequent malignancy of the urinary system, and early diagnosis is vital for effective treatment and patient prognosis 10. The effect of S100A8 on the proliferation, invasion and metastasis of BC has not been fully clarified. The present study demonstrated that S100A8 promoted BC cell proliferation, migration and invasion.

A related study has found that S100A8 and S100A9 expression is increased in gastric, lung, breast, colorectal, and prostate cancer and adenocarcinoma 32. In addition, S100A8 and S100A9 expression is downregulated in some epithelial-derived tumors, such as esophageal, uterine, and head and neck squamous cell carcinomas 33. A previous study has shown that S100A8 and S100A9 are BC-related proteins 34. Ebbing *et al.* have shown that calprotectin (a heterodimer of S100A8/S100A9) can be used as a urinary biomarker for detecting urothelial BC 35. Using magnetic bead enrichment, ion trap tandem mass spectrometry and IHC analysis of 12 invasive BC tissue biopsies and normal bladder tissue from the same patient, Tolson *et al.* reported high S100A8 expression in tumor cells in 50% of the samples compared with that in normal urothelial cells 36. Nicklas *et al.* used tissue chip technology and IHC to evaluate S100A8 protein expression 37. The authors used Kaplan-Meier survival analysis and Cox regression analysis to determine whether S100A8 expression was associated with recurrence-free, progression-free or cancer-specific survival. Their results revealed high S100A8 expression in NMIBC.

Therefore, S100A8 is a potential marker to identify patients at high risk of NMIBC recurrence and progression 37. These findings are consistent with the results of the present study. In the present study, MIBC was compared with NMIBC, and high-grade BC was compared with low-grade BC using the GEO database. The results demonstrated that S100A8 expression was significantly higher in MIBC than in NMIBC, and S100A8 expression was significantly higher in high-grade BC. The survival analysis results indicated lower overall and cancer-free survival in the high S100A8 expression group than the low S100A8 expression group. Next, GSEA of S100A8 was performed by analyzing RNA-sequencing data from the BC dataset in TCGA. The analysis revealed a significant positive association with multiple types of BC, such as stage IV and invasive BCs, suggesting that the S100A8 gene may be involved in the proliferation, invasion and migration of bladder urothelial cancer.

The present study confirmed through IHC that S100A8 protein expression was higher in BC tissues compared with adjacent tissues, higher in MIBC tissues compared with NMIBC tissues, and higher in high-grade BC tissues compared with low-grade BC tissues. Additionally, the present study confirmed by WB and RT-qPCR that the expression levels of both S100A8 protein and mRNA in BC cells were higher than those in normal urothelial cells, indicating that S100A8 expression was increased in BC cells.

Studies have demonstrated that S100A8/A9 can stimulate the proliferation, migration and invasion of most cancer types. For example, Liu *et al.* demonstrated that S100A9 promoted the phosphorylation of JNK, P38 and ERK1/2, regulating the progression, migration, invasion and metastasis in lung adenocarcinoma 38. Zheng *et al.* reported that S100A9 upregulated the phosphorylation of two important molecules, P60 and P65, in the NF-κB pathway, promoting hepatocellular carcinoma progression 39. Lv *et al.* indicated that S100A9 binds to TLR4, activating the NF-κB pathway to stimulate tumor cells to secrete fibronectin, and subsequently activating integrin β1-focal adhesion kinase, which is involved in prostate cancer cell invasion 40. Pan *et al.* revealed that, in cholangiocarcinoma cells, S100A8 upregulated VEGF expression through the TLR4-NF-κB pathway to promote the invasion and metastasis of cholangiocarcinoma cells 29. These findings are similar to the results of the present study.

The innovation of this study is to use the CCLE database to screen suitable bladder cancer cells, namely HT-1376. HT-1376 cells have higher S100A8 expression, which can better perform lentiviral knockdown and overexpression operations, and the results are more reliable. In the present study, S100A8 was overexpressed and knocked down by overexpression lentivirus and shRNA lentivirus infection of HT-1376 cells, respectively, and the infection efficiency was verified using WB and RT-qPCR. Subsequently, the effect of knockdown and overexpression on HT-1376 cell proliferation was examined using CCK-8 and EdU proliferation assays, and the effect of knockdown on HT-1376 cell invasion and migration was determined. These results demonstrated that S100A8 promoted the proliferation, invasion and migration of BC cells.

In conclusion, the present study demonstrated that S100A8 promoted the proliferation, invasion and migration of BC cells, and was highly expressed in BC. Furthermore, it was differentially expressed in NMIBC and MIBC and in high-grade and low-grade BC. Therefore, S100A8 can act as a potential biomarker for BC and enable improved diagnosis, grading and prognosis of BC. The present study explored the role of S100A8 in BC at the tissue and cellular levels; however, its role in living animals remains unclear. In subsequent studies, HT-1376 cells with overexpression and knockdown of S100A8 should be developed and injected into nude mice. Thereafter, the difference in tumor volumes in nude mice in each group should be compared. It should further be elucidated whether S100A8 can promote the development of BC *in vivo* to provide novel potential targets for the diagnosis and prognosis of BC.

## Figures and Tables

**Table 1:** Significantly different gene numbers in different bladder cancer databases

Database	High-grade vs Low-grade			MIBC vs NMIBC		
	Sig (n)	Sig.up (n)	Sig.down (n)	Sig (n)	Sig.up (n)	Sig.down (n)
GSE13507	160	88	72	156	89	67
GSE31684	1045	580	465	973	563	410
GSE120736	153	82	71	119	41	78

**Table 2:** Intersection of differentially expressed genes

Down-regulated genes				Up-regulated genes
ANXA10	SLC14A1	CDC42EP5	CAPNS2	S100A8
ST3GAL5	BTBD16	BCAS1	FAM3D	FAM83D
TMPRSS4	SPOCD1	VSIG2	HSD17B2	CDCA5
CYP3A5				

**Table 3:** GSE13507 Prognostic Meta-analysis of OS and DSS for the candidate gene list

Genes	OS			DSS	
	*P*	HR (95% *CI*)		*P*	*HR* (95% *CI*)
S100A8	0.000	1.22 [1.11 - 1.34]		0.000	1.29 [1.12 - 1.48]
CDCA5	0.001	1.36 [1.14 - 1.62]		0.000	1.83 [1.40 - 2.38]
FAM83D	0.054	1.21 [1.00 - 1.46]		0.000	1.59 [1.23 - 2.05]
FAM3D	0.006	0.77 [0.64 - 0.93]		0.001	0.60 [0.45 - 0.81]
ANXA10	0.019	0.90 [0.82 - 0.98]		0.004	0.81 [0.71 - 0.94]
SPOCD1	0.022	0.88 [0.79 - 0.98]		0.003	0.79 [0.67 - 0.92]
ST3GAL5	0.032	0.85 [0.73 - 0.99]		0.000	0.68 [0.55 - 0.84]
TMPRSS4	0.034	0.88 [0.78 - 0.99]		0.006	0.79 [0.67 - 0.93]
HSD17B2	0.120	0.91 [0.80 - 1.03]		0.011	0.80 [0.67 - 0.95]
CAPNS2	0.175	0.89 [0.76 - 1.05]		0.097	0.82 [0.65 - 1.04]
BTBD16	0.180	0.93 [0.83 - 1.03]		0.009	0.82 [0.71 - 0.95]
BCAS1	0.196	0.90 [0.76 - 1.06]		0.007	0.74 [0.59 - 0.92]
SLC14A1	0.226	0.93 [0.82 - 1.05]		0.038	0.83 [0.70 - 0.99]
CDC42EP5	0.306	0.92 [0.79 - 1.08]		0.044	0.81 [0.65 - 0.99]
VSIG2	0.511	0.95 [0.83 - 1.10]		0.016	0.81 [0.69 - 0.96]
					

**Table 4:** Correlation between S100A8 protein in bladder cancer tissue and clinical characteristics of patients

Correlative factor	The expression of the S100A8		*χ^2^*	*P*
	positive	negative		
**Age**			0.351	0.554
Below 60	10	8		
Above 60	18	10		
**Gender**			0.097	1.000
Male	24	16		
Female	4	2		
**Histological grade**			14.220	<0.001
Low-grade	9	16		
high-grade	19	2		
**Pathological stage**			19.237	<0.001
NMIBC	8	18		
MIBC	19	1		

MIBC, muscle invasive bladder cancer; NMIBC, non-muscle invasive bladder cancer; S100A8, S100 calcium-binding A8.

## Abbreviations

S100A8: S100 calcium-binding A8
BC: Bladder cancer
MIBC: Muscle invasive BC
NMIBC: Non-muscle invasive BC
GEO: Gene Expression Omnibus
TCGA: The Cancer Genome Atlas
GSEA: Gene set enrichment analysis
KEGG: model of Kyoto Encyclopedia of Genes and Genomes
qRT-PCR: quantitative reverse transcript-PCR

## References

[1] Lobo N, Shariat SF, Guo CC (2020). What is the significance of variant histology in urothelial carcinoma?. Eur Urol Focus.

[2] Yang H, Dinney CP, Ye Y, Zhu Y, Grossman HB, Wu X (2008). Evaluation of genetic variants in microRNA-related genes and risk of bladder cancer. Cancer Res.

[3] Sung H, Ferlay J, Siegel RL (2021). Global cancer statistics 2020: GLOBOCAN estimates of incidence and mortality worldwide for 36 cancers in 185 countries. CA Cancer J Clin.

[4] Zheng RS, Chen R, Han BF (2024). Cancer incidence and mortality in China, 2022. Chin J Oncol.

[5] Netto GJ, Amin MB, Berney DM (2022). The 2022 World Health Organization classification of tumors of the urinary system and male genital organs—part B: prostate and urinary tract tumors. Eur Urol.

[6] Magers MJ, Lopez-Beltran A, Montironi R, Williamson SR, Kaimakliotis HZ, Cheng L (2019). Staging of bladder cancer. Histopathology.

[7] Kandori S, Kojima T, Nishiyama H (2019). The updated points of TNM classification of urological cancers in the 8th edition of AJCC and UICC. Jpn J Clin Oncol.

[8] Liedberg F, Hagberg O, Holmäng S (2015). Local recurrence and progression of non-muscle-invasive bladder cancer in Sweden: a population-based follow-up study. Scand J Urol.

[9] Nykopp TK, Batista Da Costa J, Mannas M, Black PC (2018). Current clinical trials in non-muscle invasive bladder cancer. Curr Urol Rep.

[10] Lenis AT, Lec PM, Chamie K, Mshs MD (2020). Bladder Cancer: A Review. Jama.

[11] Zhu CZ, Ting HN, Ng KH, Ong TA (2019). A review on the accuracy of bladder cancer detection methods. J Cancer.

[12] Xing J, Reynolds JP (2018). Diagnostic advances in urine cytology. Surg Pathol Clin.

[13] Saprykina EV, Sal'nik B (1988). The role of lipid metabolism disorders in the mechanism of the hepatotoxic effects of rubomycin. Antibiot Khimioter.

[14] Guo A, Wang X, Gao L, Shi J, Sun C, Wan Z (2014). Bladder tumour antigen (BTA stat) test compared to the urine cytology in the diagnosis of bladder cancer: A meta-analysis. Can Urol Assoc J.

[15] Zippe C, Pandrangi L, Potts JM, Kursh E, Novick A, Agarwal A (1999). NMP22: a sensitive, cost-effective test in patients at risk for bladder cancer. Anticancer Res.

[16] Dimashkieh H, Wolff DJ, Smith TM, Houser PM, Nietert PJ, Yang J (2013). Evaluation of urovysion and cytology for bladder cancer detection: a study of 1835 paired urine samples with clinical and histologic correlation. Cancer Cytopathol.

[17] He H, Han C, Hao L, Zang G (2016). ImmunoCyt test compared to cytology in the diagnosis of bladder cancer: A meta-analysis. Oncol Lett.

[18] Dyrskjøt L, Hansel DE, Efstathiou JA (2023). Bladder cancer. Nat Rev Dis Primers.

[19] Grados DFW, Ahmadi H, Griffith TS, Warlick CA (2022). Immunotherapy for Bladder Cancer: Latest Advances and Ongoing Clinical Trials. Immunol Invest.

[20] Witjes JA, Bruins HM, Cathomas R (2021). European Association of Urology Guidelines on Muscle-invasive and Metastatic Bladder Cancer: Summary of the 2020 Guidelines. Eur Urol.

[21] Aveldaño MI (1995). Phospholipid solubilization during detergent extraction of rhodopsin from photoreceptor disk membranes. Arch Biochem Biophys.

[22] Odink K, Cerletti N, Brüggen J (1987). Two calcium-binding proteins in infiltrate macrophages of rheumatoid arthritis. Nature.

[23] Trøstrup H, Lerche CJ, Christophersen L, Jensen P, Høiby N, Moser C (2017). Immune modulating topical S100A8/A9 inhibits growth of pseudomonas aeruginosa and mitigates biofilm infection in chronic wounds. Int J Mol Sci.

[24] Clark HL, Jhingran A, Sun Y (2016). Zinc and manganese chelation by neutrophil S100A8/A9 (calprotectin) limits extracellular Aspergillus fumigatus hyphal growth and corneal infection. J Immunol.

[25] Ye Y, Pei L, Ding J, Wu C, Sun C, Liu S (2019). Effects of Platycodin D on S100A8/A9-induced inflammatory response in murine mammary carcinoma 4T1 cells. Int Immunopharmacol.

[26] Xiong J, Wang T, Tang H, Lv Z, Liang P (2019). Circular RNA circMAN2B2 facilitates glioma progression by regulating the miR-1205/S100A8 axis. J Cell Physiol.

[27] Koh HM, An HJ, Ko GH (2019). Prognostic role of S100A9 expression in patients with clear cell renal cell carcinoma. Medicine.

[28] Lyu ZJ, Wang Y, Huang JL (2021). Recurrent ZNF83-E293V mutation promotes bladder cancer progression through the NF-κB pathway via transcriptional dysregulation of S100A8. Mol Ther.

[29] Pan S, Hu Y, Hu M (2020). S100A8 facilitates cholangiocarcinoma metastasis via upregulation of VEGF through TLR4/NF-κB pathway activation. Int J Oncol.

[30] Nedjadi T, Evans A, Sheikh A (2018). S100A8 and S100A9 proteins form part of a paracrine feedback loop between pancreatic cancer cells and monocytes. BMC Cancer.

[31] Livak KJ, Schmittgen TD (2001). Analysis of relative gene expression data using real-time quantitative PCR and the 2(-Delta Delta C(T)) Method. Methods.

[32] Salama I, Malone PS, Mihaimeed F, Jones JL (2008). A review of the S100 proteins in cancer. Eur J Surg Oncol.

[33] Luo A, Kong J, Hu G (2004). Discovery of Ca2+-relevant and differentiation-associated genes downregulated in esophageal squamous cell carcinoma using cDNA microarray. Oncogene.

[34] Bansal N, Gupta A, Sankhwar SN, Mahdi AA (2014). Low- and high-grade bladder cancer appraisal via serum-based proteomics approach. Clin Chim Acta.

[35] Ebbing J, Mathia S, Seibert FS (2014). Urinary calprotectin: a new diagnostic marker in urothelial carcinoma of the bladder. World J Urol.

[36] Tolson JP, Flad T, Gnau V (2006). Differential detection of S100A8 in transitional cell carcinoma of the bladder by pair wise tissue proteomic and immunohistochemical analysis. Proteomics.

[37] Nicklas AP, Kramer MW, Serth J (2018). Calgranulin A (S100A8) immunostaining: A future candidate for risk assessment in patients with non-muscle-invasive bladder cancer (NMIBC). Adv Ther.

[38] Liu P, Wang H, Liang Y (2018). LINC00852 promotes lung adenocarcinoma spinal metastasis by targeting S100A9. J Cancer.

[39] Zheng H, Jiang WH, Tian T (2017). CBX6 overexpression contributes to tumor progression and is predictive of a poor prognosis in hepatocellular carcinoma. Oncotarget.

[40] Lv Z, Li W, Wei X (2020). S100A9 promotes prostate cancer cell invasion by activating TLR4/NF-κB/integrin β1/FAK signaling. Onco Targets Ther.

